# Design Analysis of Adhesively Bonded Structures

**DOI:** 10.3390/polym9120664

**Published:** 2017-12-01

**Authors:** Ee-Hua Wong, Johan Liu

**Affiliations:** 1 Sino-Singapore International Joint Research Institute, Guangzhou 510550, China; 2 Energy Research Institute, Nanyang Technological University, Nanyang 639798, Singapore; 3 Microtechnology and Nanoscience, Chalmers University of Technology, Gothenburg Se41296, Sweden; johan.liu@chalmers.se; 4 SMIT Center, Shanghai University, No 20, Chengzhong Road, Shanghai 201800, China

**Keywords:** balanced structures, unbalanced structures, single lap joint, closed-form solutions

## Abstract

The existing analytical solutions for the peeling and shearing stresses in polymeric adhesively bonded structures are either too inaccurate or too complex for adoption by practicing engineers. This manuscript presents a closed-form solution that is reasonably accurate yet simple and concise enough to be adopted by practicing engineers for design analysis and exploration. Analysis of these concise solutions have yielded insightful design guidelines: (i) the magnitude of peeling stress is generally higher than that of shearing stress; (ii) the peeling stress in a balanced structure may be reduced most effectively by reducing the elastic modulus of the adherends or by increasing the adhesive-to-adherend thickness ratio and less effectively by reducing the elastic modulus of the adhesive; and (iii) the peeling stress in an unbalanced structure may be reduced by increasing the in-plane compliance of the structure, which may be implemented most effectively by reducing the thicknesses of the adherends and less effectively by reducing the elastic modulus of the adherends.

## 1. Introduction

The polymeric adhesive in many bonded structures is much more structurally compliant than the adherends, leading to a significantly lower magnitude of in-plane stress in the adhesive. Completely ignoring this in-plane stress in the adhesive substantially reduces the complexity of the analysis and allows the formulation of closed-form solutions. The first of such analyses was presented by Volkersen (1938) [1], who modelled the adhesive layer in a single lap-shear structure as having only shear stiffness and the adherends as capable of only in-plane stretching. A more sophisticated analysis was presented by Goland and Reissner (1944) [2], who modelled the adhesive as having stiffness in shearing and transverse stretching and the adherends as capable of in-plane stretching and bending.

Goland and Reissner [2] assumed the adherends to have negligible shear and transverse-normal compliances, which may not be valid for bonded structures with relatively large ratios of adherends-to-adhesive thicknesses. Assuming a linear distribution of shear stress along the thickness of the adherends—An overly simplistic assumption—Tsai et al. (1998) [3] incorporated shear compliance of the adherends into the formulation of Goland and Reissner [2]. But before Tsai et al. [3], Suhir (1986, 1989) has evaluated the shear compliance [4] and the transverse compliance [5] of the adherends using Ribiere Solution for a long-and-narrow strip [6].

Besides assuming nil in-plane stress in the adhesive, the above authors and many others [7,8,9,10,11,12] have also conveniently assumed that the shear and the transverse stresses are unvarying over the thickness of the adhesive. Ojalo and Eidinoff (1978) [13] were believed to be the first to challenge this assumption; assuming linear variation of the in-plane and the transverse deformations of the adhesive along its thickness, they concluded that the shear stress varies linearly while the transverse stress is unvarying along the thickness of the adhesive. More recently, Wang and Zhang (2009) [14] assumed that transverse stress in the adhesive exhibits a step-jump in magnitude while shear stress is unvarying along its thickness. These assumptions invariably violate the differential equation of equilibrium:

(1) $$\begin{array}{l} \frac{\partial \sigma_{x}}{\partial x}+\frac{\partial \tau_{xz}}{\partial z}=0\\ \frac{\partial \sigma_{z}}{\partial z}+\frac{\partial \tau_{xz}}{\partial x}=0 \end{array}$$

Except for [14], these strength-of-material solutions have also failed to satisfy the condition that there shall be no shear stress at the free edges of the adhesive, thus leading to gross underestimation of the magnitude of peeling stress at the free edge [15].

Moreover, the strength-of-material solution of Goland and Reissner [2] was restricted to balanced structures—That is, structures in which the adherends are identical in geometry and materials—For which the resultant differential equations for the shear and the transverse stresses in the adhesive are uncoupled and can be solved with relative ease. Delale et al. (1981) [8], Bigwood and Crocombe (1989) [9], Liu et al. (2014) [11], Zhao et al. (2011) [16], and Zhang et al. (2015) [17] have analysed unbalanced structures experiencing arbitrary edge loading such that the differential equations are heavily coupled. The resultant closed-formed solutions are immensely chunky and complex.

A “theory of elasticity” solution that is based on variational formulation was presented by Allman (1977) [18]. The solution satisfied Equation (1) while also ensuring that the free-edge condition of nil-shear stress was satisfied. Similar approach was followed by Chen and Cheng (1983) [19], Yin (1991) [20], Adams and Mallick (1992) [21], and Wu and Zhao (2013) [22]. The solutions are not only complex but highly restrictive—The boundary conditions are embedded within the governing differential equation, thus limiting the generality of the solutions. 

From the perspective of practicing engineers, the current state of closed-form solutions for adhesively bonded structures is far from satisfactory—These solutions are either too inaccurate or too complex for practical adoption. It is the objective of this manuscript to offer a closed-form solution that is reasonably accurate yet simple and concise enough to be adopted by practicing engineers for design analysis and exploration.

## 2. Analytical Equations

**Figure 1:**
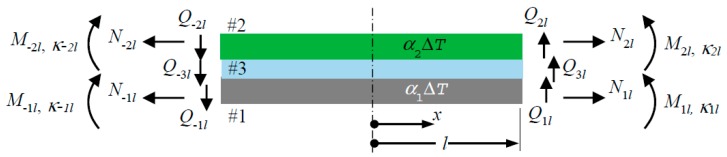
Schematic of a bonded structure experiencing general conditions of edge loading and thermal strain.

Figure 1 shows a bonded structure made up of adherend #1, adherend #2, and adhesive #3. The structure experiences a mismatched thermal expansion between the adherends and stretching, shearing, and bending at their edges. Note the notations and positive directions of the in-plane stretching forces *N*_±*il*_, the shear forces *Q*_±*il*_, moments *M*_±*il*_, and curvatures *κ*_±*il*_, at *x* = ±*l*, where *l* is the half-length of the adhesive. The height, Young’s modulus, shear modulus, flexural stiffness, and thermal coefficient of expansion of member #*i* are denoted as *h_i_*, *E_i_*, *G_i_*, *D_i_*, and *α_i_*, respectively. The shear, in-plane (*x*), transverse (*z*), and flexural compliances of the bonded structure are denoted as *κ_s_*, *λ_x_*, *λ_z_*, and $\bar{D}$, respectively. The corresponding compliances of member #*i* are denoted as *κ_si_*, *λ_xi_*, *λ_zi_*, and $\bar{D_{i}}$, respectively. The formulas for computing these compliances are collected under the heading “basic formula” at the front of this article. The derivations of these formulas may be found in Refs. [15,23].

The adhesive is assumed to experience negligible in-plane stress, *σ_x_*. The differential equation of equilibrium, Equation (1), then suggests an nonvarying shear stress, *τ_xz_*, and a linearly varying transverse stress, *σ_z_*, along the thickness of the adhesive. Denoting *σ_m_* and *σ_a_* respectively as the mean and amplitude of variation of the transverse stress along the thickness of the adhesive, the peeling stress at the two interfaces of the adhesive with adherend #*i* is given by

(2) $$\sigma_{pi}=\sigma_{m}\mp \sigma_{a},\;i=1,2$$

**Figure 2:**
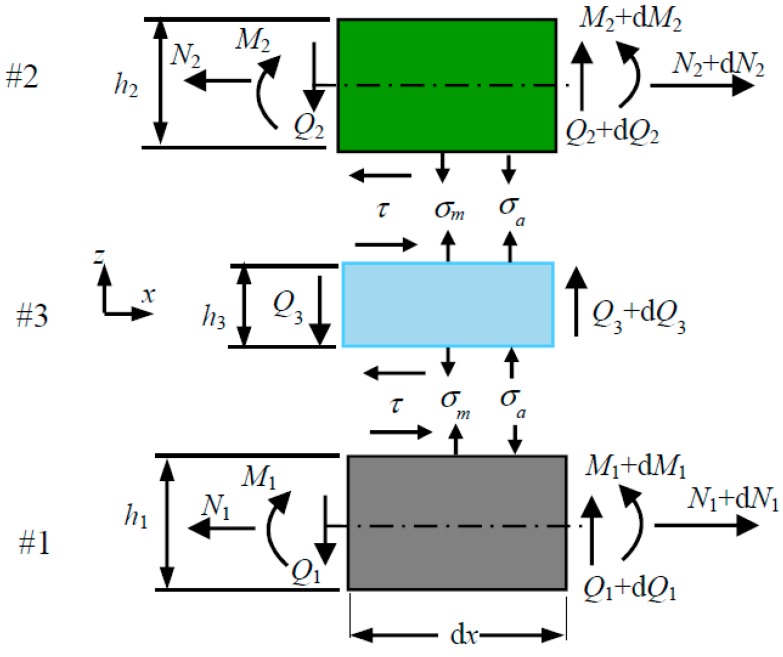
Elemental representation of a bonded structure.

Unless otherwise stated in this manuscript, the upper and the lower signs in ∓ are associated with adherend #1 and adherend #2 respectively. These stresses, together with the interfacial shear stress, *τ*, on an elemental representation of a bonded structure are shown in Figure 2. Substituting *∂σ_z_*/*∂z* as *σ_a_*/(2*h*_3_) into Equation (1) gives

(3) $$\sigma_{a}=-\frac{h_{3}}{2}\frac{d\tau }{dx}$$

The derivations of the differential equations for *τ* and *σ_m_* are elaborated upon in Appendix A.

### 2.1. Balanced Structures

Referring to Equation (A5) and with the parameter, *µ_σ_*, equates to nil for a balanced bonded structure, the differential equation for the interfacial shear stress is given by

(4) $$\frac{d^{3}\tau }{dx^{3}}-\beta^{2}\frac{d\tau }{dx}=0$$

where $\beta^{2}=\lambda_{x}/\kappa_{s}$. For structures of reasonably large length, say *βl* > 3, the solution is given approximately by

(5) $$\tau (x)=A_{c}e^{\beta (x-l)}+A_{3},\;x>\;0$$

The boundary conditions *κ_s_dτ*(l)/*dx* = *ε_T_* + *ε_Nl_* + *ε_Ml_* and $\int_{-l}^{l}\tau dx=N_{2l}-N_{-2l}$ return

(6) $$\begin{array}{l} A_{\pm c}=\frac{\varepsilon_{T}+\varepsilon_{\pm Nl}+\varepsilon_{\pm Ml}}{\beta \kappa_{s}}\\ A_{3}=\frac{N_{2l}-N_{-2l}}{2l}-\frac{A_{c}-A_{-c}}{2\beta l}=\frac{N_{2l}-N_{-2l}}{2l}-\frac{\varepsilon_{Nl}-\varepsilon_{-Nl}+\varepsilon_{Ml}-\varepsilon_{-Ml}}{2\lambda_{x}l} \end{array}$$

where

(7) $$\begin{array}{l} \varepsilon_{T}=\left(\alpha_{2}-\alpha_{1}\right)∆T\\ \varepsilon_{\pm Nl}=N_{\pm 2l}\lambda_{x2}-N_{\pm 1l}\lambda_{x1}\\ \varepsilon_{\pm Ml}=\frac{1}{2}\left(\left(h_{1}+h_{3}\right)\kappa_{\pm 1l}+\left(h_{2}+h_{3}\right)\kappa_{\pm 2l}\right) \end{array}$$

Referring to Equation (A6) and with the parameter, *µ_τ_*, equates to nil for a balanced bonded structure, the differential equation for the mean of the peeling stress is given by

(8) $$\frac{d^{4}\sigma_{m}}{dx^{4}}+4\alpha^{4}\sigma_{m}=0$$

where $4\alpha^{4}=\bar{D}/\lambda_{z}$. For structures of reasonably large length, say *αl* > 3, the solution is given approximately by

(9) $$\sigma_{m}(x)=e^{\alpha (x-l)}\left[B_{1c}\cos\alpha (x-l)+B_{2c}\sin\alpha (x-l)\right],\;x\;>\;0.$$

The boundary conditions $\lambda_{z}d^{2}\sigma_{m}/dx^{2}=M_{2l}/D_{2}-M_{1l}/D_{1}={\hat{M}}_{21l}$ and $\lambda_{z}d^{3}\sigma_{m}/dx^{3}=-(Q_{2l}/D_{2}-Q_{1l}/D_{1})=-{\hat{Q}}_{21l}$ return

(10) $$B_{1c}=\frac{{\hat{Q}}_{21l}}{2\alpha^{3}\lambda_{z}}+B_{2c},B_{2c}=\frac{{\hat{M}}_{21l}}{2\alpha^{2}\lambda_{z}}$$

Substituting Equation (5) into Equation (3) yields the amplitude of the peeling stress:

(11) $$\sigma_{a}(x)=-\frac{A_{s}\beta h_{3}}{2}e^{\beta (x-l)}\;\mathrm{for}\;x\;>\;0.$$

### 2.2. Unbalanced Structures

#### 2.2.1. Non-Free Edge Solutions

Combining Equations (A5) and (A6) gives rise to a seventh-order differential equation for *τ*(*x*) and a sixth-order differential equation for *σ_m_*(*x*) [5,7,8,9,11]. The resulting solutions are far too complex to be attractive to practicing engineers. Instead, solving Equations (A5) and (A6) iteratively would lead to approximate solutions of *τ*(*x*) and *σ_m_*(*x*) that are far simpler, thus enabling insights and encouraging application by practicing engineers. Designating the subscripts *c* and *p* as complimentary and particular solutions, respectively, the approximate solution involves solving for the complementary solutions for the interfacial shear stress, *τ_c_*, and the mean of the peeling stress, *σ_mc_*—these are given by Equations (5) and (9)—followed by substituting *σ_mc_* into Equation (A5) to obtain *τ* =*τ_c_* + *τ_p_*, and *τ_c_* into Equation (A6) to obtain *σ_m_* = *σ_mc_* + *σ_mp_*.

The interfacial shear and peel stresses have been evaluated as

(12) $$\begin{array}{l} \tau (x)=A_{s}e^{\beta (x-l)}+A_{4}+e^{\alpha (x-l)}\left[A_{p1}\cos\alpha (x-l)+A_{p2}\sin\alpha (x-l)\right]\\ \sigma_{m}(x)=e^{\alpha (x-l)}\left[B_{1}\cos\alpha (x-l)+B_{2}\sin\alpha (x-l)\right]+B_{p}e^{\beta (x-l)}\\ \sigma_{a}(x)=-\frac{h_{3}}{2}\left[\beta A_{s}e^{\beta (x-l)}+\alpha e^{\alpha (x-l)}\left[\left(A_{p1}+A_{p2}\right)\cos\alpha (x-l)-\left(A_{p1}-A_{p2}\right)\sin\alpha (x-l)\right]\right] \end{array}$$

where

(13) $$\begin{array}{l} A_{s}=A_{c}-\frac{\alpha \left(A_{p1}+A_{p2}\right)}{\beta }\\ A_{4}=A_{3}-\frac{1}{2l}\left(\frac{1}{2\alpha }-\frac{\alpha }{\beta^{2}}\right)\left(A_{p1}-A_{-p1}\right)+\frac{1}{2l}\left(\frac{1}{2\alpha }+\frac{\alpha }{\beta^{2}}\right)\left(A_{p2}-A_{-p2}\right)\\ A_{\pm p1}=\frac{\mu_{\sigma }}{\kappa_{s}}\frac{\left(2\alpha^{3}+\alpha \beta^{2}\right)B_{\pm 1c}+\left(2\alpha^{3}-\alpha \beta^{2}\right)B_{\pm 2c}}{2\alpha^{2}\left(4\alpha^{4}+\beta^{4}\right)},\;A_{p2}=\frac{\mu_{\sigma }}{\kappa_{s}}\frac{\left(2\alpha^{3}+\alpha \beta^{2}\right)B_{\pm 2c}-\left(2\alpha^{3}-\alpha \beta^{2}\right)B_{\pm 1c}}{2\alpha^{2}\left(4\alpha^{4}+\beta^{4}\right)}\\ B_{\pm 1c}=\frac{{\hat{Q}}_{\pm 21l}}{2\alpha^{3}\lambda_{z}}+\;B_{\pm 2c},B_{\pm 2c}=\frac{{\hat{M}}_{\pm 21l}}{2\alpha^{2}\lambda_{z}}\\ B_{1}=\frac{\beta^{3}B_{p}+{\hat{Q}}_{21l}/\lambda_{z}}{2\alpha^{3}}+B_{2},B_{2}=\frac{-\beta^{2}B_{P}+{\hat{M}}_{21l}/\lambda_{z}}{2\alpha^{2}}\\ B_{P}=\frac{\mu_{\tau }A_{c}}{\lambda_{z}}\frac{\beta }{4\alpha^{4}+\beta^{4}} \end{array}$$

It is worth noting that in the case of an unbalanced bonded structure experiencing only mismatched thermal expansion and/or differential stretching between the adherends, the magnitude of *σ_m_* is negligibly small compared to that of *τ* and *σ_a_* [23]. Ignoring *σ_m_*, the differential equation for *τ* is given by Equation (4); and *τ*(*x*) and *σ_a_*(*x*) are given by Equations (5) and (11), respectively.

#### 2.2.2. Free Edge Solutions

The expression of shear stress in Equation (12) does not satisfy the free edge condition, *τ*(*l*) = 0, which is essential for accurate modelling of *σ_a_*(*l*). The free edge condition may be enforced artificially through the introduction of a decay function, 1 − *e^nβ^*^(*x*−*l*)^ [15,23]. The derivation of the factor *n* is explained in Appendix B. The expressions of *τ*(*x*), *σ_m_*(*x*), and *σ_a_*(*x*) with the free edge condition enforced are given by

(14) $$\tau (x)=\left\{A_{s}e^{\beta (x-l)}+A_{4}+e^{\alpha (x-l)}\left[A_{p1}\cos\alpha (x-l)+A_{p2}\sin\alpha (x-l)\right]\right\}\left[1-e^{n\beta (x-l)}\right]$$

(15) $$\begin{array}{ll} \sigma_{m}(x) & =e^{\alpha (x-l)}\left[B_{1n}\cos\alpha (x-l)+B_{2n}\sin\alpha (x-l)\right]+B_{p}e^{\beta (x-l)}-B_{pn1}e^{\left(n+1)\beta (x-l\right)}-B_{pn2}e^{n\beta (x-l)}\\ & \approx e^{\alpha (x-l)}\left[B_{1n}\cos\alpha (x-l)+B_{2n}\sin\alpha (x-l)\right]+B_{p}e^{\beta (x-l)} \end{array}$$

(16) $$\sigma_{a}(x)=-\frac{h_{3}}{2}\left\{\begin{array}{l} \left(1-e^{n\beta (x-l)}\right)\left\{\beta A_{s}e^{\beta (x-l)}+\alpha e^{\alpha (x-l)}\left[\left(A_{p1}+A_{p2}\right)\cos\alpha (x-l)-\left(A_{p1}-A_{p2}\right)\sin\alpha (x-l)\right]\right\}-\\ n\beta e^{n\beta (x-l)}\left\{A_{s}e^{\beta (x-l)}+A_{4}+e^{\alpha (x-l)}\left[A_{p1}\cos\alpha (x-l)+A_{p2}\sin\alpha (x-l)\right]\right\} \end{array}\right\}$$

where

(17) $$\begin{array}{l} B_{1n}=\frac{\beta^{3}\left(B_{p}-{\left(n+1\right)}^{3}B_{pn1}-n^{3}B_{pn2}\right)+{\hat{Q}}_{21l}/\lambda_{z}}{2\alpha^{3}}+B_{2n},B_{2n}=\frac{-\beta^{2}\left(B_{p}-{\left(n+1\right)}^{2}B_{pn1}-n^{2}B_{pn2}\right)+{\hat{M}}_{21l}/\lambda_{z}}{2\alpha^{2}}\\ B_{pn1}=\frac{\mu_{\tau }A_{c}}{\lambda_{z}}\frac{(n+1)\beta }{4\alpha^{4}+{\left(n+1\right)}^{4}\beta^{4}},B_{pn2}=\frac{\mu_{\tau }A_{3}}{\lambda_{z}}\frac{n\beta }{4\alpha^{4}+n^{4}\beta^{4}} \end{array}$$

## 3. Numerical Validations

**Figure 3:**
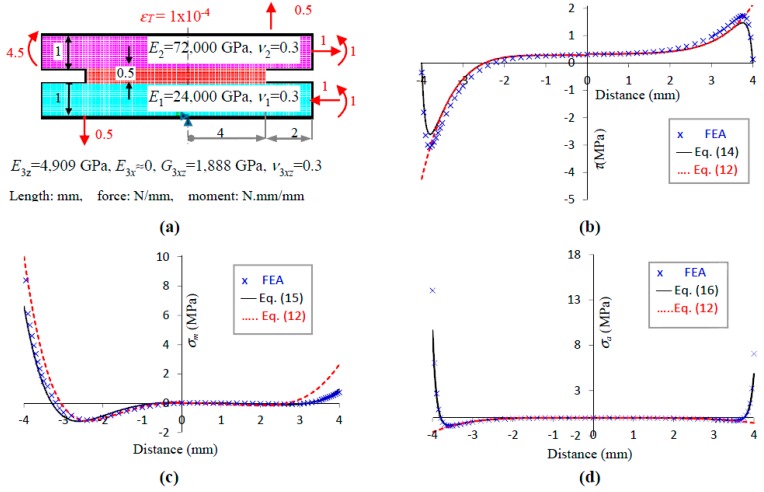
Numerical validation: (**a**) model; (**b**) *τ*(*x*); (**c**) *σ_m_*(*x*) and (**d**) *σ_a_*(*x*).

Equations (5), (9), and (11) for the balanced structures [15] and for unbalanced bonded structure experiencing only mismatched thermal expansion and/or differential stretching between the adherends [23] have been validated in previous publications. Hence, only the equations for unbalanced structures experiencing the general state of edge loading shall be validated. The material properties, dimensions of the bonded structure, thermomechanical loads, and finite element model used in this study are shown in Figure 3a. The domain of the bonded structure was modelled using more than 100,000 eight-node quadrilateral elements. The domain around the free edge was discretised at 75 divisions per mm. The adhesive was assigned with anisotropic properties of negligible *E_x_*, rendering it consistent with the assumption in the analytical solutions that the adhesive experiences negligible *σ_x_*. The compliances (assuming plane stress), characteristic parameters (*α*, *β*), free-edge parameters (*ϕ*, *n*) which are computed using Equations (A9) and (A10), coupling parameters (*µ_σ_*, *µ_τ_*), and the coefficients in the stress equations are tabulated in Table 1. It is noted that (i) *βl*, *αl* ≥ 4, thus justifying the use of the reduced form of solutions as presented in Section 2; (ii) the magnitudes of the coupling parameters are significantly large, reflecting the significant difference in the stiffness between the two adherends; and (iii) the relatively strong coupling has led to the relatively large magnitudes of the particular stress coefficients (*A_p_*_1_, *A_p_*_2_, *B_p_*) compared to that of the complementary stress coefficients (*A_s_*, *B*_1n_, *B_2n_*).

Figure 3b shows the shear stress, *τ*(*x*), for (i) the analytical solution that enforces the nil-shear stress free-edge condition, Equation (14), (ii) the analytical solution that does not enforce the above free-edge condition, Equation (12), and (iii) the finite element analysis (FEA) solution. The analytical solution, Equation (14), agrees reasonably well with the FEA solution for the entire length of the bonding although the magnitude of the former is consistently at approximately 85% that of the FEA. The free-edge parameter at *ϕ* = 0.45 is in reasonable agreement with that extracted from FEA at *ϕ_FEA_ =* 0.40 and 0.48 for *−l* ≤ *x* ≤ 0 and *0* ≤ *x* ≤ −*l*, respectively.

Figure 3c shows the mean of the transverse stress, *σ_m_*(*x*), for (i) the analytical solution that enforces the nil-shear stress free-edge condition, Equation (15), (ii) the analytical solution that does not enforce the above free-edge condition, Equation (12), and (iii) the FEA solution. The free-edge solution, Equation (15), agrees well with the FEA solution, especially if the FEA solutions at *x =* ±*l*, which are susceptible to numerical error, are ignored.

Figure 3d shows the amplitude of the transverse stress, *σ_a_*(*x*), for (i) the analytical solution that enforces the nil-shear stress free-edge condition, Equation (16), (ii) the analytical solution that does not enforce the above free-edge condition, Equation (12), and (iii) the FEA solution. Both Equation (16) and the FEA show the magnitude of *σ_a_* increases rapidly near the free edge. In contrast, Equation (12) shows a very mild increase in magnitude near the free edge, in the opposite direction.

## 4. Design Analysis

### 4.1. Balanced Structures

The shear stress in the adhesive for a balanced structure is given by Equation (5). For simplicity, we shall assume *A_c_* » *A*_3_, the maximum magnitude of shear stress is given by

(18) $$\tau_{\max}\approx A_{c}=\frac{\varepsilon_{T}+\varepsilon_{Nl}+\varepsilon_{Ml}}{\sqrt{\lambda_{x}\kappa_{s}}}$$

For the same magnitude of the applied strains, the magnitude of shear stress may be reduced by increasing the *x*-compliance and the shear compliances of the bonded structure. We shall analyse two extreme cases; case I: the ratio *h*_adhesive_*/h*_adherend_ is significant such that the shear compliance of the structure is dominated by that of the adhesive; that is, *κ_s_* ≈ *h*_3_/*G*_3_, and case II: *h*_adhesiv*e*_*/h*_adherend_ is insignificantly small, such that the shear compliance of the structure is dominated by that of the adherends; that is, *κ_s_* ≈ *h*_1_/4*G*_1_. The product *λ_x_κ_s_* for the two extreme cases (for plane stress) are given by

(19) $$\lambda_{x}\kappa_{s}=\{\begin{array}{cc} \frac{2h_{3}/h_{1}}{E_{1}G_{3}}\left(4+3h_{3}/h_{1}\right) & \mathrm{Case}\;I\\ \frac{2}{E_{1}G_{1}} & \mathrm{Case}\;\mathrm{II} \end{array}$$

Thus, for Case I, the magnitude of shear stress in the adhesive may be reduced by reducing the elastic moduli of the adherends and the adhesive while increasing the thickness ratio of adhesive-to-adherend. For Case II, the magnitude of shear stress in the adhesive may be reduced by reducing the elastic modulus of the adherends.

The mean of the transverse stress in the adhesive, *σ_m_*, for a balanced structure is given by Equation (9). The maximum magnitude of *σ_m_* occurs at *x* = *l* and is given by

(20) $$\sigma_{m,\max}\;=B_{1c}=\frac{1}{2\alpha^{2}\lambda_{z}}\left(\frac{{\hat{Q}}_{21l}}{\alpha }+{\hat{M}}_{21l}\right)$$

Noting that 2*α*^2^*λ_z_* = $\sqrt{\lambda_{z}\bar{D}}$, ${\hat{Q}}_{21l}=Q_{2l}/D_{2}-Q_{1l}/D_{1}$, ${\hat{M}}_{21l}=M_{2l}/D_{2}-M_{1l}/D_{1}$ and *D*_1_ = *D*_2_, for the same magnitude of the normalised edge forces, (*Q*_2*l*_ − *Q*_1*l*_)/*α*, and the edge moments, *M*_2*l*_-*M*_1*l*_, the magnitude of *σ_m,_*_max_ may be reduced by increasing the product *λ_z_*$\bar{D}$*D*_1_^2^ = 2*λ_z_D*_1_; that is, by increasing the *z-*compliance and the flexural stiffness of the structure. We shall analyse the same two extreme cases described for shear stress : *λ_z_* ≈ *h*_3_/*E*_3_ for Case I and *λ_z_* ≈ 13*h*_1_/16*E*_1_ for Case II. The product *λ_z_D*_1_ for the two extreme cases (for plane stress) are given by

(21) $$\lambda_{z}D_{1}=\{\begin{array}{c} \frac{h_{1}{}^{3}h_{3}E_{1}/E_{3}}{12}\;\mathrm{Case}\;I\\ \;\frac{13h_{1}{}^{4}}{192}\;\mathrm{Case}\;\mathrm{II} \end{array}$$

Thus, for Case I, the magnitude of *σ_m,_*_max_ may be reduced most effectively by increasing the thickness of the adherends and less effectively by increasing the thickness of the adhesive or the ratio of *E*_adherend_-to-*E*_adhesive_. For Case II, the magnitude of *σ_m,_*_max_ may be reduced very effectively by increasing the thickness of the adherends.

The amplitude of the transverse stress in the adhesive, *σ_a_*, for a balanced structure is given by Equation (11), which however ought to be modified to include the free-edge condition:

(22) $$\sigma_{a}(x)=-\frac{h_{3}}{2}\left\{\left(1-e^{n\beta (x-l)}\right)\beta A_{c}e^{\beta (x-l)}-n\beta e^{n\beta (x-l)}\left[A_{c}e^{\beta (x-l)}+A_{3}\right]\right\}$$

The maximum magnitude of *σ_m_* occurs at *x* = *l*. For simplicity, we shall assume *A_c_* » *A*_3_; the maximum magnitude of *σ_a_*, after substituting the exponential factor *n* with Equation B(2) is given approximately by

(23) $$\sigma_{a,\max}\approx \frac{n\beta h_{3}A_{c}}{2}\approx \frac{0.72A_{c}}{\phi^{1.4}{\left(\beta h_{3}\right)}^{0.4}}\approx \frac{0.72}{\phi^{1.4}}\frac{\varepsilon_{T}+\varepsilon_{Nl}+\varepsilon_{Ml}}{\sqrt[{4}]{\lambda_{x}{}^{3}\kappa_{s}h_{3}{}^{2}}}$$

Assuming *ϕ* to be a constant, then for the same magnitudes of the applied strains, the magnitude of *σ_a,_*_max_ may be reduced by increasing the product *λ_x_*^3^*κ_s_h*_3_^2^, which for the two extreme cases: *κ_s_* ≈ *h*_3_/*G*_3_ for Case I and *κ_s_* ≈ *h*_1_/4*G*_1_ for Case II - are given by (for plane stress)

(24) $$\lambda_{x}{}^{3}\kappa_{s}h_{3}{}^{2}=\{\begin{array}{c} \frac{8{\left(h_{3}/h_{1}\right)}^{3}{\left(4+3h_{3}/h_{1}\right)}^{3}}{E_{1}{}^{3}G_{3}}\;\mathrm{Case}\;I\\ \frac{128{\left(h_{3}/h_{1}\right)}^{2}}{E_{1}{}^{3}G_{1}}\;\mathrm{Case}\;\mathrm{II} \end{array}$$

Thus, for Case I, the magnitude of *σ_a,_*_max_ may be reduced most effectively by reducing the elastic modulus of the adherends or by increasing the thickness ratio of adhesive-to-adherend and less effectively by reducing the elastic modulus of the adhesive. For Case II, the magnitude of *σ_m,_*_max_ may be reduced most effectively by reducing the elastic modulus of the adherends and less effectively by increasing the thickness ratio of adhesive-to-adherend.

It is clear from the above that the design guidelines do not concurrently minimise *τ*_max_, *σ_a_*_,max_, and *σ_m_*_,max_. In other words, the design guidelines for minimizing the magnitudes of *τ*_max_, *σ_a_*_,max_, and *σ_m_*_,max_ are contradictory. We shall examine the relative magnitudes of these components of stress. Equation (25) presents the ratio of *σ_a_*_,max_/*τ*_max_. It is noted that the magnitude of *σ_a_*_,max_ is almost always larger than that of *τ*_max_. This, together with the fact that the peeling strength of a bonded joint is generally weaker than its shear strength, suggests that it is more important to minimise the magnitudes of *σ_a_*_,max_ and *σ_m_*_,max_ than the magnitude of *τ*_max_.

(25) $$\begin{array}{ll} \frac{\sigma_{a,\max}}{\tau_{\max}} & =\frac{n\beta h_{3}}{2}\approx \frac{0.72}{\varphi^{1.4}{\left(\beta h_{3}\right)}^{0.4}}\approx \frac{2}{{\left(\beta h_{3}\right)}^{0.4}}\;\mathrm{assuming}\;\varphi =0.5\\ & \approx 2{\left[\frac{E_{1}h_{1}}{2G_{3}h_{3}\left(4+3h_{3}/h_{1}\right)}\right]}^{0.2}\;\mathrm{for}\;\mathrm{Case}\;I\;\\ & \approx 3\;\mathrm{to}\;4\;\mathrm{for}\;E_{1}h_{1}/G_{3}h_{3}\;\mathrm{between}\;60\;\mathrm{to}\;250 \end{array}$$

Equation (26) presents the ratio of *σ_a_*_,max_/*σ_m_*_,max_. Noting that *σ_m_*_,max_ ≈ 0 for adherends experiencing only thermal strain and/or stretching strain, *σ_a_*_,max_ is much larger than *σ_m_*_,max_ for these cases. Noting that *σ_a_*_,max_ ≈ 0 for adherends experiencing only edge shear forces, *Q_il_*, *σ_a_*_,max_ is much smaller than *σ_m_*_,max_ for this case. Thus, *σ_a_*_,max_ may be larger or smaller than *σ_m_*_,max_ for a general loading condition. For the case of single lap-joints (SLJ) that are dominated by stretching and bending through a single adherend, i.e., *ε_T_* = 0, ${\hat{Q}}_{21l}\;\approx \;0$, *κ*_1*l*_ = 0, while *N*_2*l*_ and *M*_2*l*_ are positive, *σ_a_*_,max_ is almost always larger than *σ_m_*_,max_. It is therefore advisable to give more weight to the design guidelines that minimize the magnitude of *σ_a_*_,max_.

(26) $$\begin{array}{ll} \frac{\sigma_{a,\max}}{\sigma_{m,\max}} & =\frac{n\beta h_{3}}{2}\sqrt{\frac{\lambda_{z}\bar{D}}{\lambda_{x}\kappa_{s}}}\frac{\varepsilon_{T}+\varepsilon_{Nl}+\varepsilon_{Ml}}{{\hat{Q}}_{21l}/\alpha +{\hat{M}}_{21l}}\\ & \approx \frac{n\beta h_{3}}{4}\sqrt{\frac{6}{\left(4+3h_{3}/h_{1}\right)\left(1+\nu \right)}}\left(\frac{N_{2l}h_{1}}{6M_{2l}}+1+h_{3}/h_{1}\right)>1\;\mathrm{for}\;\mathrm{SLJ}\;\mathrm{and}\;\mathrm{Case}\;I \end{array}$$

### 4.2. Unbalanced Structures

In light of the coupling of *τ*(x) and *σ_m_*(*x*) for the unbalanced structures, it is improbable to find a universal guideline for the optimum design of unbalanced structures that can be expressed in the simple forms of Equations (19), (21) and (24).

The shear stress in the adhesive of an unbalanced structure is given by Equation (14). For simplicity, we shall assume *A_s_* » *A*_4_ while also ignoring the decay function, 1 − *e^nβ^*^(*x−l*)^, the maximum magnitude of shear stress is then given by

(27) $$\begin{array}{ll} \tau_{\max} & \approx A_{s}+A_{p1}=A_{c}-\frac{\alpha }{\beta }\left(A_{p1}+A_{p2}\right)+A_{p1}\\ & =\frac{\varepsilon_{T}+\varepsilon_{Nl}+\varepsilon_{Ml}}{\sqrt{\lambda_{x}\kappa_{s}}}+\frac{\mu_{\sigma }\left[\left(2\alpha^{3}-2\alpha^{2}\beta +\alpha \beta^{2}\right){\hat{Q}}_{21l}/\alpha +\left(-4\alpha^{4}/\beta +4\alpha^{3}-2\alpha^{2}\beta \right){\hat{M}}_{21l}\right]}{4\alpha^{4}\left(4\alpha^{4}+\beta^{4}\right)\lambda_{z}\kappa_{s}} \end{array}$$

The magnitude of *A_c_* may be reduced by increasing the *x-*compliance and the shear compliance of the structure. Equation (27) suggests that the magnitude of the particular solution may be reduced by increasing the product $\left(4\alpha^{4}+\beta^{4}\right)\lambda_{z}\kappa_{s}$, which implies increasing the *x-*compliance and the flexural compliance of the structure. While increasing the *x-*compliance of the structure would reduce the magnitudes of both the complimentary and particular solutions, this does not imply that it will always lead to reduced magnitude of the *τ*_max_ of the unbalanced structure. This is because *ε_T_*, *ε_Nl_*, *ε_Ml_*, ${\hat{Q}}_{21l}$, ${\hat{M}}_{21l}$ and *µ_σ_* could be either positive or negative and hence the particular solution may not act in the same direction as the complementary solution. Nevertheless, increasing the *x-*compliance of the structure is more likely than not to reduce the *τ*_max_ of an unbalanced structure. In the same breath, it should be noted that the magnitude of *τ*_max_ in an unbalanced structure may not always be larger than that in a balanced structure assuming both structures have identical characteristic parameters. 

The mean of the transverse stress in the adhesive, *σ_m_*, for an unbalanced structure is given by Equation (15). For the reason of simplicity, we shall ignore those terms associated with the decay function. The maximum magnitude of *σ_m_* occurs at *x* = *l* and is given by

(28) $$\begin{array}{ll} \sigma_{m,\max} & \approx B_{1}+B_{p}=B_{1c}+\left(\frac{\beta^{3}}{2\alpha^{3}}-\frac{\beta^{2}}{2\alpha^{2}}+1\right)B_{p}\\ & =\frac{1}{\sqrt{\bar{D}\lambda_{z}}}\left[\frac{{\hat{Q}}_{21l}}{\alpha }+{\hat{M}}_{21l}+\frac{\mu_{\tau }\left(\beta^{4}/\alpha -\beta^{3}+2\alpha^{2}\beta \right)}{4\alpha^{4}+\beta^{4}}\frac{\varepsilon_{T}+\varepsilon_{Nl}+\varepsilon_{Ml}}{\beta \kappa_{s}}\right] \end{array}$$

Noting that ${\hat{Q}}_{21l}=Q_{2l}/D_{2}-Q_{1l}/D_{1}$ and ${\hat{M}}_{21l}=M_{2l}/D_{2}-M_{1l}/D_{1}$, for the same magnitudes of *Q_il_* and *M_il_*, the magnitude of *B*_1_ may be reduced by increasing the *z-*compliance while reducing the flexural compliance of the structures. Equation (28) suggests that the magnitude of the particular solution may be reduced by increasing the magnitudes of the characteristic parameter, *α*, which implies increasing the flexural compliance of the structure while reducing the *z-*compliance of the structure. This is in reverse to the trend for *B_p_*. 

The amplitude of the transverse stress in the adhesive, *σ_a_*, for an unbalanced structure is given by Equation (16). Assuming *A_s_* » *A*_4_, the maximum magnitude of *σ_a_* is given by:

(29) $$\sigma_{a,\max}\approx \frac{n\beta h_{3}\left(A_{s}+A_{p1}\right)}{2}\approx \frac{n\beta h_{3}}{2}\tau_{\max}\approx \frac{0.72}{\varphi^{1.4}{\left(\beta h_{3}\right)}^{0.4}}\tau_{\max}$$

which again suggests that the magnitude of *σ_a,_*_max_ is likely to be reduced by increasing the *x-*compliance of the structure.

It has been argued in the previous section that the magnitude of *σ_a_*_,max_ is almost always larger than that of *τ*_max_ for balanced structures. Comparing Equations (23) and (29), it is reasonably safe to suggest that this will also be the case for unbalanced structures. It has been established in the previous section that the ratio of *σ_a,_*_max_ to *σ_m,_*_max_ for balanced SLJ is larger than unity. If the ratio for the particular solutions is also larger than unity then one can safely assume that the ratio of *σ_a,_*_max_ to *σ_m,_*_max_ for unbalanced SLJ is also larger than unity. The ratio for the particular solutions is given by

(30) $$\frac{\sigma_{a,\max}}{\sigma_{m,\max}}\approx \frac{n\beta h_{3}}{2}\frac{\mu_{\sigma }\left(-2\alpha^{2}/\beta +2\alpha -\beta \right)\kappa_{2l}}{\mu_{\tau }\left(\beta^{3}/\alpha -\beta^{2}+2\alpha^{2}\right)\frac{\varepsilon_{Nl}+\varepsilon_{Ml}}{\sqrt{\lambda_{x}\kappa_{s}}}}$$

which is not necessary larger than unity. Thus, *σ_a,_*_max_ is only conditionally larger than *σ_m,_*_max_ for unbalanced SLJ.

Given the loadings and the design space, the optimum designs of unbalanced structure that gives rise to a minimum *τ*_max_, a minimum *σ_m,_*_max_, and a minimum *σ_a,_*_max_, respectively, can be readily established using Equations (27)–(29).

## 5. Conclusions

Strength-of-material solutions for the shear stress, *τ*, the mean, *σ_m_*, and amplitude, *σ_a_*, of the peeling stress in both balanced and unbalanced structures have been derived and used for design analysis. Design guidelines for balanced structures have been established. The magnitude of *σ_a,_*_max_ for balanced structures is almost always larger than that of *τ*_max_ and the magnitude of *σ_a,_*_max_ for balanced single-lap-joints is also almost always larger than *σ_m,_*_max_. The magnitude of *σ_a,_*_max_ for balanced structures may be reduced most effectively by reducing the elastic modulus of the adherends or by increasing the thickness ratio of adhesive-to-adherend and less effectively by reducing the elastic modulus of the adhesive. The simple expressions of *τ*_max_, *σ_m,_*_max_ and *σ_a,_*_max_ established in this manuscript for the unbalanced structures will help practicing engineers find the optimum design within the given design space.

## Figures and Tables

**Table 1:** Numerical validation: characteristics of the unbalanced structure.

Compliances		Characteristic parameters (mm^−1^)				Free edge parameters	
*κ_s_*	2.83 × 10^−4^	*α*		1.08		*ϕ*	0.45
*λ_x_*	4.31 × 10^−4^	*β*		1.23		*n*	7.4
*λ_z_*	1.21 × 10^−4^	Coupling parameters (N^−1^)					
		*µ_σ_*		1.67 × 10^−4^		*µ_τ_*	2.50 × 10^−4^
Stress coefficients (N. mm^−2^)							
Domain	*A_s_*	*A*_4_	*A*_*p*1_	*A*_*p*2_	*B*_1*n*_	*B*_2*n*_	*B*_*p*_
*−l *≤ *x* ≤ 0	3.23	0.31	1.33	0.83	4.94	7.23	1.67
0 ≤ *x* ≤ l	1.53		0.3	0.1	0.35	0.98	0.61

*κ_s_*, *λ_z_* (N^−1^.mm^3^), *λ_x_* (N^−1^.mm).

## Nomenclatures

Subscript #*_i_*	Subscript #1 and #2 are adherends and #3 is adhesive.
*D*_i_, *E*_i_, *G*_i_, *h*_i_	Flexural rigidity, elastic modulus, shear modulus, thickness of member #*i*.
$\bar{D}$	Flexural compliance of the bonded structure.
*M*_±*il*_, *N*_±*il*_, *Q*_±*il*_	Moment, sectional stretching force, sectional shear force applied on adherend #*i* at *x* = ±*l*.
*l*	Half-length of the bonded structure.
*α*, *β*	Characteristic parameters of a bonded structure in peeling, shearing.
*α_i_*	Coefficient of thermal expansion of adherend #*i*.
*ε_T_*, *ε*_±*Nl*_, *ε*_±*Ml*_	Differential strain between adherends #2 and #1 at *x* = ±*l* due to temperature and edge stretching; effective bending strain due to edge bending at *x* = ±*l*.
*κ*_±*il*_	Edge curvature of adherend #*i* at *x* = ±*l*.
*κ_si_, κ_s_*	Shear compliance of member #*i*, of the bonded structure between the centroid planes of adherends #1 and #2.
*λ_xi_, λ_x_*	*x*-compliance of adherend #*i*, of the bonded structure.
*λ_xθ_*	Additional *x*-compliance of the bonded structure attributed to its flexural deformation.
*λ_zi_, λ_z_*	*z*-compliance of member #*i*, of the bonded structure between the centroid planes of adherends #1 and #2.
*θ_i_*	Rotation of the centroid axis of adherend #*i* (due to bending).
*σ_m_*(*x*), *σ_a_*(*x*)	Mean, amplitude of variation, of transverse stress along the thickness of the adhesive (or between the bonded interfaces).
*σ_p_*(*x*)	Peeling stress along the bonded interfaces.
*τ*(*x*)	Shear stress within the adhesive (and along the interfaces).
Δ*T*	Temperature change.
**Basic formulas**	
$4\alpha^{4}=\frac{\bar{D}}{4\lambda_{z}}$; $\beta^{2}=\frac{\lambda_{x}}{\kappa_{s}}$.	
$D_{i}=\frac{E_{i}h_{i}{}^{3}}{12}$(plane stress); $\bar{D}=\frac{1}{D_{1}}+\frac{1}{D_{2}}$.	
$\kappa_{s}=\kappa_{s1}+\kappa_{s2}+\;\kappa_{s3}$	
$\kappa_{si}\approx \frac{h_{i}}{8G_{i}},i=1,2;\kappa_{s3}=\frac{h_{3}}{G_{3}}$	
$\lambda_{x}=\lambda_{x1}+\lambda_{x2}+\lambda_{x\theta }$	
$\lambda_{xi}=\frac{1}{E_{i}h_{i}},\;i=1,2;\;\lambda_{x\theta }=\frac{1}{4}\left[\frac{h_{1}\left(h_{1}+h_{3}\right)}{D_{1}}+\frac{h_{2}\left(h_{2}+h_{3}\right)}{D_{2}}\right]$	
$\lambda_{z}=\lambda_{z1}+\lambda_{z2}+\lambda_{z3}$	
$\lambda_{zi}\approx \;\frac{13}{32}\frac{h_{i}}{E_{i}},\;i=1,2;\lambda_{z3}=\frac{h_{3}}{E_{3}}$	
$\mu_{\sigma }=\frac{1}{2}\left(\frac{h_{2}}{D_{2}}-\frac{h_{1}}{D_{1}}\right),\mu_{\tau }=\frac{1}{2}\left(\frac{h_{2}+h_{3}}{D_{2}}-\frac{h_{1}+h_{3}}{D_{1}}\right)$	

## Appendix A. Fundamental Equations

Refers to Figure 2, assuming the traction *N_i_* passes through the centroid plane of adherend #1, the equilibriums of the differential elements gives

(A1) $$\begin{array}{l} dN_{i}=\mp \tau dx\\ dQ_{i}=\left(\mp \sigma_{m}+\sigma_{a}\right)dx\\ dQ_{3}=-2\sigma_{a}dx\;i=1,\;2\\ dM_{i}=\left(\frac{\tau h_{i}}{2}-Q_{i}\right)dx \end{array}$$

where the upper and the lower signs in “∓” refers to adherend #1 and #2, respectively. The *x*-directional traction-stretching relation of the centroid plane of adherend #*i* is given by:

(A2) $$\frac{du_{i}}{dx}-\alpha_{i}∆T=N_{i}\lambda_{xi}$$

The moment-rotation relation of the centroid plane of adherend #*i* is assumed to obey simple beam:

(A3) $$\frac{d^{3}w_{i}}{dx^{3}}=\frac{d^{2}\theta_{i}}{dx^{2}}=\frac{1}{D_{i}}\frac{dM_{i}}{dx}=\frac{1}{D_{i}}\left(\frac{h_{i}\tau }{2}-Q_{i}\right)$$

The differential displacement of the centroid planes of adherend #1 and #2 in the *u*-direction and in the *z*-direction are given by:

(A4) $$\begin{array}{l} u_{2}-u_{1}=\kappa_{s}\tau -\frac{h_{1}\theta_{1}+h_{2}\theta_{2}}{2}\\ w_{2}-w_{1}=\lambda_{z}\sigma_{m} \end{array}$$

where *θ_i_* is the rotation of the neutral axis of member #*i*. Differentiating Equation (A2) twice with respect to *x* followed by taking the difference between adherend #2 and #1, and equating with the third differential of the first equation of (A4) gives the differential equation:

(A5) $$\frac{d^{3}\tau }{dx^{3}}-\beta^{2}\frac{d\tau }{dx}=-\frac{\mu_{\sigma }}{k_{s}}\sigma_{m}$$

Differentiating Equation (A3) with respect to *x* followed by taking the difference between adherends #2 and #1, and equating with the fourth differential of the second equation of (A4) gives the differential equation

(A6) $$\frac{d^{4}\sigma^{m}}{dx^{4}}+4\alpha^{4}\sigma^{m}=\frac{\mu_{\tau }}{\lambda_{z}}\frac{d\tau }{dx}$$

The parameters and the coefficients are given by

(A7) $$\begin{array}{l} \beta^{2}=\frac{\lambda_{x}}{\kappa_{s}},\;4\alpha^{4}=\frac{\bar{D}}{\lambda_{z}}\\ \lambda_{x}=\lambda_{x1}+\lambda_{x2}+\sum_{i=1}^{2}\frac{h_{i}(h_{i}+h_{3})}{4D_{i}}\\ \kappa_{s}=\sum_{i=1}^{3}\kappa_{si},\;\lambda_{z}=\sum_{i=1}^{3}\lambda_{zi},\;\overset{¯}{D}=\sum_{i=1}^{2}\frac{D_{i}}{1}\\ \mu_{\sigma }=\frac{1}{2}(\frac{h_{2}}{D_{2}}-\frac{h_{1}}{D_{1}}),\;\mu_{\tau }=\frac{1}{2}(\frac{h_{2}+h_{3}}{D_{2}}-\frac{h_{1}+h_{3}}{D_{1}}) \end{array}$$

## Appendix B. Imposing Free-Edge Condition

The free-edge condition, *τ*(*l*) = 0, can be artificially imposed by multiplying the expression of the shear stress, Equation (12), with a decay function 1−*e*^*nβ*(*x*−1)^, wherein *n* is a positive real number with a magnitude significantly larger than unity such that the decay function diminishes rapidly from the free edge towards *x* = 0. Assuming (i) *τ_c_(x)* ≈ *A_c_e^β(x−l)^*(1−*e^nβ(x−l)^*) and (ii) the stationary point of *τ*(*x*) occurs at a distance *ϕ**h*_3_ from the free edge, where *ϕ* is assumed to be a constant, the magnitude of *n* may be evaluated approximately by differentiating *τ_c_*(*x*) with respect to *x* followed by equating the stationary point with *l*−*φh*_3_. This yields:

*n = e^nϕβh_3_^* − 1 ≈ 1.43(*ϕβh_3_*)^−1.40^ (A8)

which for ease of engineering manipulation may be approximated as

*n* ≈ 1.43(*ϕβh_3_*)^−1.40^ (A9)

The “constant” *ϕ* is approximately 0.5 but has been established through collocating with the finite element analysis as:

*ϕ* ≈ 0.407(βh_3_^0.88^)^−0.26^ A(10)

for 0.018 ≤ *βh*_3_ ≤ 1.73.
